# Giant Mucocele of the Remnant Esophagus: Case Report of a Rare Complication Following a Bipolar Esophageal Exclusion Procedure

**DOI:** 10.7759/cureus.6317

**Published:** 2019-12-07

**Authors:** Saravanan Manickam Neethirajan, Chandramohan S M, Vaithiswaran Velayoudam, Lakshman Aridhasan Meenakshi, Sakthivel Harikrishnan

**Affiliations:** 1 Institute of Gastrosciences, Gleneagles Global Health City, Chennai, IND; 2 Institute of Gastrosciences, Glenegales Global Health City, Chennai, IND; 3 Surgical Gastroenterology, Madras Medical College, Chennai, IND; 4 Surgical Gastroenterology and Liver Transplant, Government Stanley Medical College, Chennai, IND

**Keywords:** esophageal mucocele, esophageal exclusion

## Abstract

We describe a case of a symptomatic mucocele of the esophagus following surgical isolation of the diseased esophagus, which needed surgical resection. A 33-year-old male presented to us with shock, high-grade fever, and breathlessness five days after the onset of sudden, severe lower chest and upper abdominal pain preceded by an episode of retching and vomiting. He was initially managed elsewhere by right intercostal drainage for right-sided pleural effusion, broad-spectrum parenteral antibiotics, and total parenteral nutrition. CT chest showed a right loculated pleural effusion and distal oesophageal perforation with active contrast leak into the right pleural space. He was subsequently referred to us in view of suspected Boerhaave’s syndrome and clinical worsening. In view of hemodynamic instability with uncontrolled sepsis, he was planned for surgery. Intraoperatively, there was a 4 cm long distal oesophageal perforation, 4 cm above the esophagogastric junction on the right, with an unhealthy apex, communicating with a large abscess cavity in the right pleural space with thick purulent contents. End cervical esophagostomy with esophagogastric junction stapling and feeding jejunostomy was performed in addition to transhiatal drainage of the abscess at the lower end of the esophagus and the placement of an additional intercostal drain. The postoperative period was uneventful, and he was discharged. After two months, he was assessed for possible esophagectomy and gastric pullup. Dense adhesions at thoracoscopy precluded any esophageal delineation and dissection. Attempted transhiatal dissection of the esophagus was unsuccessful in view of cicatrization, and it was decided to forego esophagectomy and proceed with bypass alone by a retrosternal gastric pull-up and cervical esophagogastrostomy. He was discharged following an uneventful postoperative period of recovery. Three months later, the patient presented with complaints of pain in the chest for three weeks, associated with hiccups. He was diagnosed to have a mucocele of the remnant esophagus based on a CT scan. The esophageal mucocele was excised by a transthoracic approach and, he was relieved of the pressure symptoms.

Following the esophageal exclusion procedure, a mucocele of the remnant esophagus can develop due to the accumulation of secretions leading to subsequent dilatation. Small mucoceles are usually asymptomatic and often go unnoticed. However, in rare cases, it may enlarge to cause compression symptoms such as respiratory distress, chest pain, cough, hiccups, and an inability to swallow. Cross-sectional imaging clinches the diagnosis, and definitive surgery consists of surgical resection by a transthoracic approach.

## Introduction

A symptomatic mucocele of the remnant esophagus is a rare complication following bipolar esophageal exclusion procedures. Mucoceles occur due to the collection of secretions within the esophageal blind loop. They are usually self-limited, and there is no established theory to explain this self-limited nature of esophageal mucoceles. However, the most widely accepted mechanism for this self-regulation of the size of mucoceles is that persistent secretion from the glands causes the pressure within the lumen to increase and thereby causes atresia of the glands [1]. Occasionally, they may enlarge enough to produce symptoms such as respiratory distress, chest pain, cough, hiccoughs, and dysphagia due to the compression of nearby structures. We are presenting a case of symptomatic mucocele following an esophageal exclusion procedure in a case of Boerhaave syndrome.

## Case presentation

A 33-year-old South Indian male presented with sudden-onset breathlessness and chest pain. He was evaluated outside and found to have Boerhaave syndrome. Initially, the patient was managed conservatively and then referred five days after the onset of initial symptoms to our center for further management. Physical examination revealed that the patient was febrile with mild dehydration and tachypnea. With further investigations, he was diagnosed with Boerhaave syndrome with uncontrolled sepsis. In view of uncontrolled sepsis, he was taken up for emergency laparotomy. He underwent diversion cervical esophagostomy and esophagogastric junction stapling with feeding jejunostomy.

Two months later, he was planned for the restoration of esophageal continuity but in view of dense adhesions in the mediastinum on thoracoscopy, retrosternal gastric pull-up and esophagogastric anastomosis were done by a left cervical incision. Transhiatal esophagectomy was deferred. The postoperative period was uneventful.

**Figure 1:**
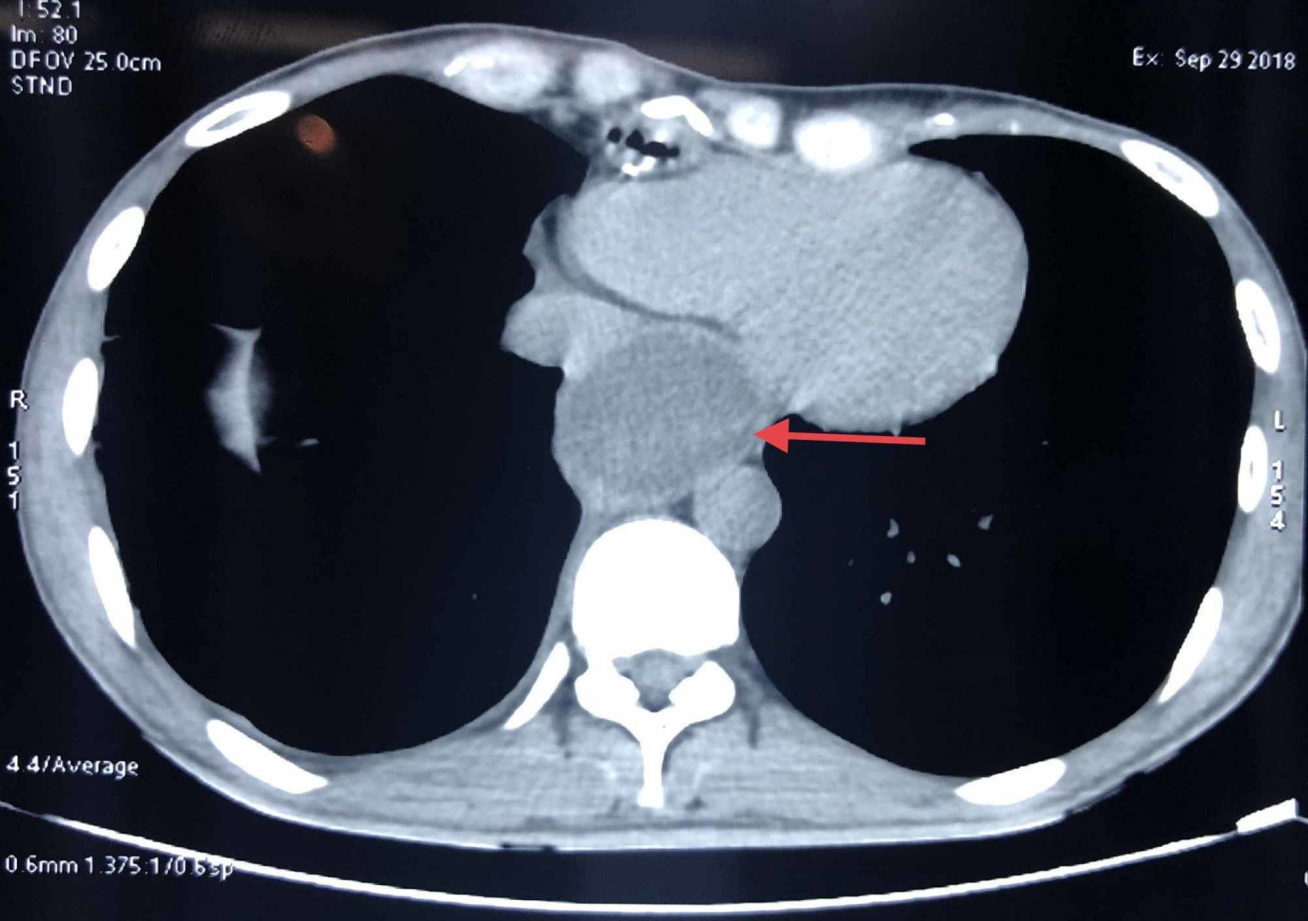
CT thorax showing the mucocele of the remnant esophagus

Three months following the gastric pull-up procedure, the patient presented with complaints of chest pain, dysphagia, and hiccoughs. There was a weight loss of 5 kilograms in the past three months. Contrast-enhanced computed tomography (CT) of the thorax revealed dilatation of the esophagus, suggestive of a mucocele (Figure 1).

**Figure 2:**
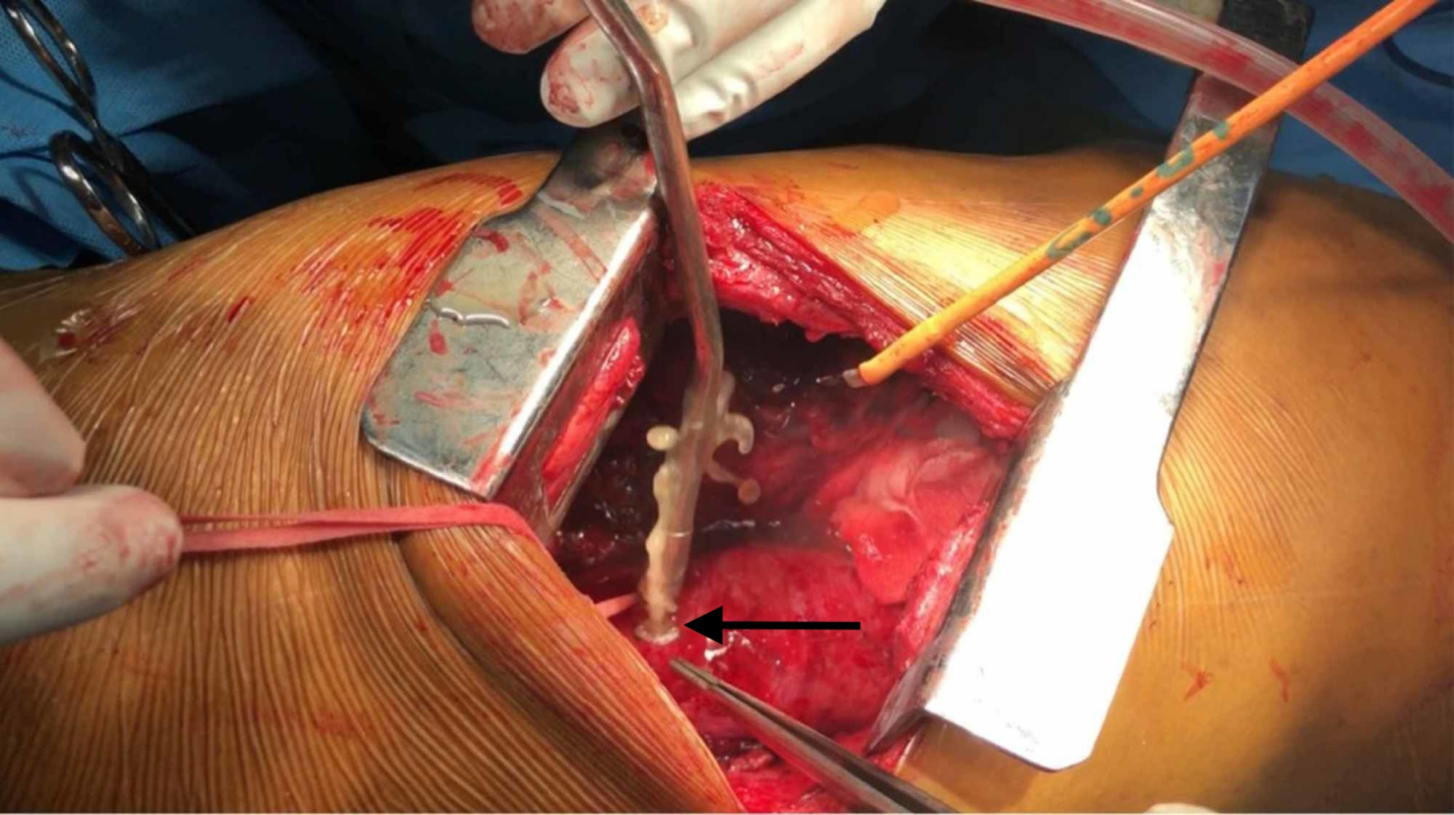
Drainage of the mucocele with a suction catheter

He was diagnosed with a mucocele of the remnant esophagus. In view of the symptomatic mucocele, he was planned for surgery. He underwent right posterolateral thoracotomy with mucocele drainage and remnant esophagectomy. The contents of the mucocele were drained and sent for culture, which reported positive for Candida species (Figure 2).

**Figure 3:**
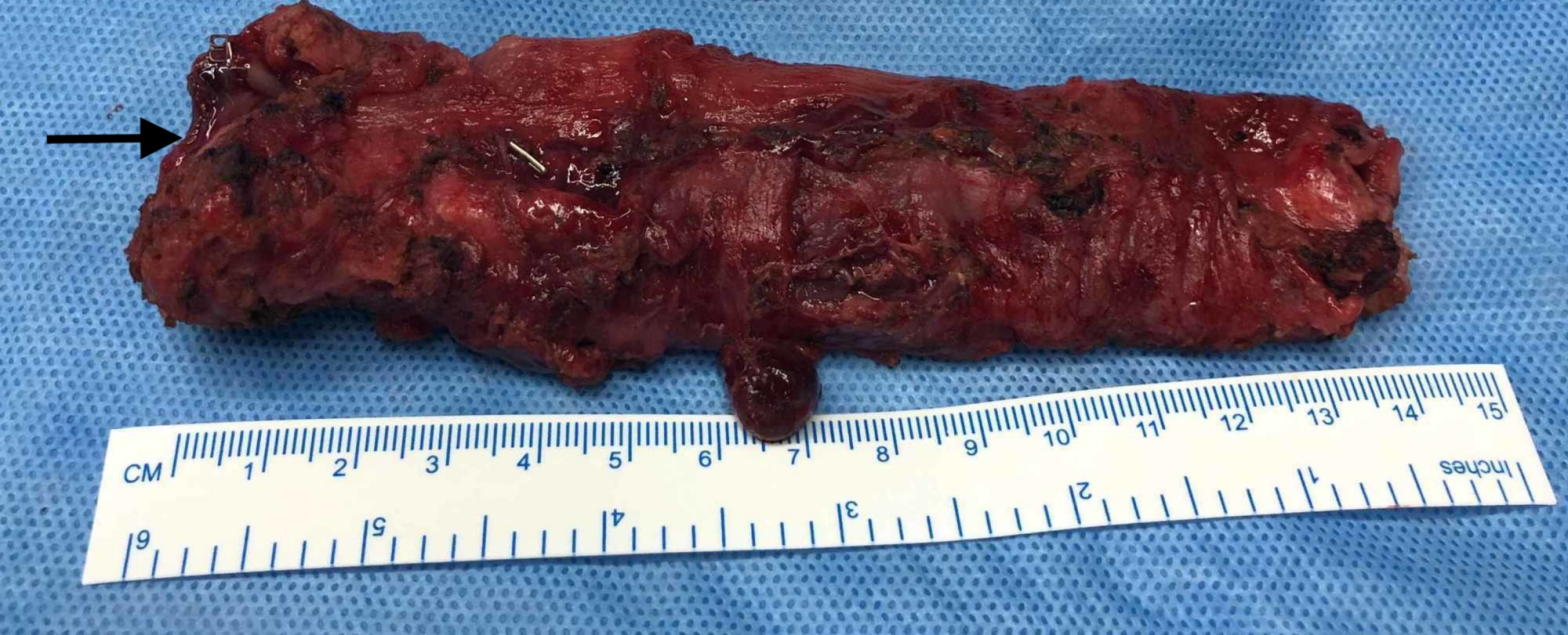
Resected specimen of the remnant esophagus

The resected specimen is shown in Figure 3.

The postoperative period was uneventful and the patient was discharged on the sixth postoperative day.

## Discussion

Spontaneous transmural esophageal perforation, also known as Boerhaave syndrome, was first reported by a German doctor, Herman Boerhaave in 1724 [2]. The prognosis of Boerhaave syndrome depends on how early the diagnosis is made and treatment is initiated. The symptoms include vomiting, chest pain (seen in 70% of the patients), dyspnea, dysphagia, subcutaneous emphysema, tachypnea, tachycardia, fever, and epigastric pain. However, these symptoms are not specific to Boerhaave syndrome and hence lead to the delay in diagnosis, which is responsible for the high mortality rate (between 10% to 25% when treated within 24 hours and between 40% and 60% when treated after 48 hours) [3]. Treatment includes volume replacement, broad-spectrum antibiotics, and surgical management. Early perforations (diagnosed within 12-24 hours) are taken up for primary surgical repair either through thoracotomy or through video-assisted thoracoscopic surgery (VATS). Late perforations (diagnosed later than 24 hours) are managed by esophagostomy with feeding gastrostomy, and esophageal replacement is done usually six weeks later [4].

When esophagectomy cannot be done, an esophageal exclusion procedure is done. Mucoceles in the excluded esophagus is a rare but reported complication of the exclusion procedure. Though the incidence of asymptomatic mucoceles following the esophageal exclusion procedure in patients with unresectable esophageal cancer, extensive corrosive injury to the esophagus, and achalasia complicated by an esophageal stricture was found to be 16% and 39% in two different studies, the occurrence of a symptomatic mucocele is rare [5-6]. Most of the symptomatic mucoceles have presented within two months of the esophageal exclusion. CT can be a very useful tool in clinching the diagnosis. A mucocele appears as a thin-walled cystic lesion in the location of the native esophagus in contrast-enhanced CT [7].

Haddad et al. reported two cases of esophageal mucoceles in patients who had previously undergone retrosternal gastric bypass procedure for caustic esophagitis and esophageal rupture respectively. Both the patients were treated with esophageal excision via a right posterolateral thoracotomy [1]. Genc et al. reported a case of invasive adenocarcinoma within an infected mucocele [8]. Collins et al. reported a case of recurrent mucocele in a patient who was treated for a bronchoesophageal fistula with an esophageal exclusion and colonic interposition. The patient was finally managed by CT-guided aspiration and ethanol ablation of the cavity [9]. We have presented a case of mucocele in a patient with Boerhaave syndrome managed with an esophageal exclusion procedure.

## Conclusions

In conclusion, it is essential that patients are surgically treated promptly when mucoceles become symptomatic, in order to prevent the formation of complications such as fistula formation, mucocele infection, and carcinomatous changes. Considering the fact that a variety of treatment options are available, such as thoracoscopic or transthoracic esophagectomy or CT-guided aspiration with ethanol ablation of the mucosa, treatment needs to be individualized according to each patient.
